# Coculturing of *Mucor plumbeus* and *Bacillus subtilis* bacterium as an efficient fermentation strategy to enhance fungal lipid and gamma-linolenic acid (GLA) production

**DOI:** 10.1038/s41598-022-17442-2

**Published:** 2022-07-30

**Authors:** Hassan Mohamed, Mohamed F. Awad, Aabid Manzoor Shah, Beenish Sadaqat, Yusuf Nazir, Tahira Naz, Wu Yang, Yuanda Song

**Affiliations:** 1 Colin Ratledge Center of Microbial Lipids, School of Agriculture Engineering and Food Science, Shandong University of Technology, Zibo, 255000 China; 2 Department of Botany and Microbiology, Faculty of Science, Al-Azhar University, Assiut, 71524 Egypt; 3 Department of Biology, College of Science, Taif University, P.O. Box 11099, Taif, 21944 Saudi Arabia; 4 Department of Food Sciences, Faculty of Science and Technology, Universiti Kebangsaan Malaysia, 43600 UKM Bangi, Selangor Malaysia

**Keywords:** Biotechnology, Chemical biology, Microbiology

## Abstract

This study aimed to improve lipid and gamma-linolenic acid (GLA) production of an oleaginous fungus, *Mucor plumbeus,* through coculturing with *Bacillus subtilis* bacteria, optimising the environmental and nutritional culture conditions, and scaling them for batch fermentation. The maximum levels of biomass, lipid, fatty acid, and GLA in a 5 L bioreactor containing cellobiose and ammonium sulfate as the optimal carbon and nitrogen sources, respectively, achieved during the coculturing processes were 14.5 ± 0.4 g/L, 41.5 ± 1.3, 24 ± 0.8, and 20 ± 0.5%, respectively. This strategy uses cellobiose in place of glucose, decreasing production costs. The nutritional and abiotic factor results suggest that the highest production efficiency is achieved at 6.5 pH, 30 °C temperature, 10% (v/v) inoculum composition, 200 rpm agitation speed, and a 5-day incubation period. Interestingly, the GLA concentration of cocultures (20.0 ± 0.5%) was twofold higher than that of monocultures (8.27 ± 0.11%). More importantly, the GC chromatograms of cocultures indicated the presence of one additional peak corresponding to decanoic acid (5.32 ± 0.20%) that is absent in monocultures, indicating activation of silent gene clusters via cocultivation with bacteria. This study is the first to show that coculturing of *Mucor plumbeus* with *Bacillus subtilis* is a promising strategy with industrialisation potential for the production of GLA-rich microbial lipids and prospective biosynthesis of new products.

## Introduction

When lipids comprise more than 20% of a microbe’s biomass, they are characterised as oleaginous microorganisms^1^. When microbial growth is unrestricted by deficiencies in carbon, nitrogen, or other nutritional sources, lipids are synthesised for energy storage^2,3^. Numerous microorganisms, such as microalgae, yeasts, and filamentous fungi, can produce more than 70% lipids with dry biomass^4^. Oleaginous microorganisms have several advantages over plants for lipid production, including short life cycles, lower demands on space, ease of scale-up, and noncompeting food sources^5^. Some lipids of microbial origin include uncommon fatty acids (FAs) such as essential FAs (EFAs), an important food supplement, and those necessary for human health, including docosahexaenoic acid (DHA), gamma-linolenic acid (GLA), eicosapentaenoic acid (EPA), and arachidonic acid (ARA)^6^.

The axenic culturing of microorganisms under laboratory conditions does not resemble their normal environment, where they are subject to extensively microbial competition. Therefore, naturally occurring stimulants and signal molecules used for chemical communication and interactions among the various organisms are absent^7^, resulting in the silencing of biosynthetic gene clusters and simplified metabolite approaches^8^. Cocultivation is a recent branch of culturing where two or more microbes with the same nutritional and abiotic environments are cultured together. It is considered an efficient strategy in many biological fields, particularly fungal research, that has successfully improved lipid production in most oleaginous filamentous fungi^9,10^. A major consequence of fungal and bacterial coculturing is high competition for the limited nutrients in the culturing media, an important ecological factor that triggers secondary metabolite biosynthesis and accumulation in both prokaryotes and eukaryotes^11^. Therefore, coculturing strategies have repeatedly led to the identification of new compounds that were not detected in individual cultures^12^.

Coculturing approaches and diverse microbial communities have been metabolically developed for purposes such as secondary metabolites, biofuels, and the production of natural products. These strategies, particularly those involving genetic modification, may reduce metabolism because metabolic pathway complexes divert resources to significantly enhance the production of the target products^13^. Coculturing of various organisms is increasingly used in microbial natural product research and metabolism in place of monocultures because the interaction of two or more organisms can improve the accumulation of constitutively present and valuable products^14^ or trigger the expression of fungal and bacterial silent biosynthetic pathways that yield novel chemical compounds^15^. Coculturing of two different microalgae provides clues to overcoming such difficulties by not causing large modifications and changes in FA composition suitable to meet demands for biodiesel^10^. A similar study investigated coculturing of *Rhodotorula glutinis* and *Chlorella vulgaris* to maximise the production of dry mass up to 4.63 g/L and lipid accumulation up to 2.88 g/L compared to monocultures^16^. In addition, the coimmobilized culturing of *C. sorokiniana* and *C. vulgaris* with *Azospirillum brasilense* significantly increased lipid concentrations to 350 mg/g cell dry weight (CDW) and the number of FA varieties from five to eight^17^.

Akone et al. previously reported that five new fungal products and two non-expressed compounds, which are absent in fungal monocultures, accumulated within cocultures of *Chaetomium* spp. fungus with *B. subtilis* bacterium^18^. Moreover, it was recently demonstrated that biomass and lipid levels increased with coculturing microalga *Euglena gracilis* with growth-promoting bacterium *Emticicia* sp. EG3^19^. Oleaginous filamentous fungi have been cocultured with microalgae to increase lipid ratios, including marine microalgae *Nannochloropsis oceanica* with the oleaginous fungus *Mortierella elongata* to initiate bioflocculation that provided maximum quantities of triacylglycerols (TAGs) and polyunsaturated FAs (PUFAs) relative to total lipid yield^20^. Interestingly, the FA composition of fermented soybean products was improved with the coculturing of the fungus *Rhizopus oligosporus* and bacteria *B. subtilis*^21^. Nevertheless, coculturing *Aspergillus nidulans* with actinobacteria *Streptomyces rapamycinicus* successfully demonstrated the molecular basis of the induction of silent biosynthetic gene clusters^22^. Therefore, coculturing has been improved and broadly used in the study of value-added products and secondary metabolites of microbial origin^23^ and is a promising strategy for increasing lipid production in industrial applications.

In this study, we cocultured *B. subtilis* ATCC 6633 with *M. plumbeus* 6697.A for the first time and assess their potential to improve fungal biomass, lipid accumulation, and GLA yield. The differences and similarities between the FA profiles in cocultures and monocultures are investigated using the submerged fermentation technology and gas chromatography (GC) analyses.

## Results

### Coculturing conditions of tested fungal strains

**Figure 1:**
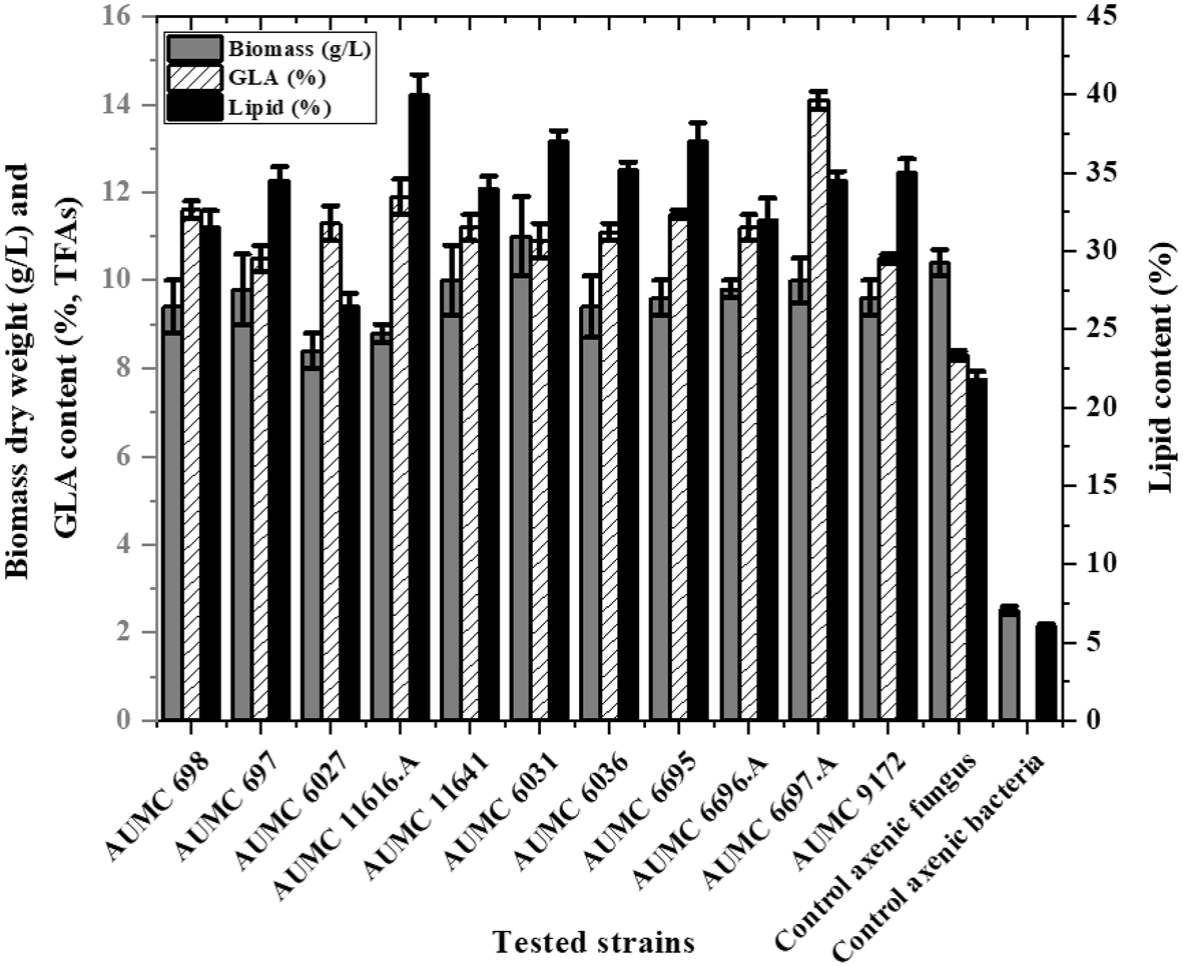
Fungal biomass, lipids, and GLA yields of assessed fungal strains cocultured with *B. subtilis* ATCC 6633 in K&R fermentation media for 4 days. Error bars represent the standard deviation (SD) of three biological replicates.

Cocultures of 11 fungi with *B. subtilis* ATCC 6633 were propagated at 10% (*v/v*; 10^7^ fungal spores/mL and 10^4^ bacterial colony-forming units [CFU]) in baffled flasks containing 150 mL Kendrick and Ratledge (K&R) fermentation media. All cocultures (11 flasks plus axenic fungal and bacterial monoculture controls) were incubated for 96 h at 30 °C with shaking at 150 rpm. The resulting biomass, lipid, and GLA production of all cocultures are shown in Fig. 1. Maximum GLA yield was obtained with *M. plumbeus* 6697.A reaching 14.1 ± 0.2% of total FAs (TFAs) in coculture compared to its monoculture control (8.3 ± 0.1%). The lowest GLA yield was obtained with *M. circinelloides* AUMC 697 (10.5 ± 0.1%) and *M. hiemalis* AUMC 9172 (10.6 ± 0.2%) compared to their monoculture controls (7.87 ± 0.1% and 9.81 ± 0.1%, respectively). The GLA production by cocultured *M. plumbeus* 6697.A was ~ twofold more than that of its monoculture control. Maximum biomass and lipid yields were 11 ± 0.3 g/L and 40.7 ± 1.4%, respectively, obtained with *M. hiemalis* AUMC 6031 and *R. pusillus* AUMC 11,616.A, and the lowest biomass and lipid yields were 8.42 ± 0.3 g/L and 26.5 ± 1.1%, respectively, obtained with *M. circinelloides* AUMC 6027. Other fungal strains assessed for their biomass, lipid, and GLA yields are shown in Fig. 1.

**Figure 2:**
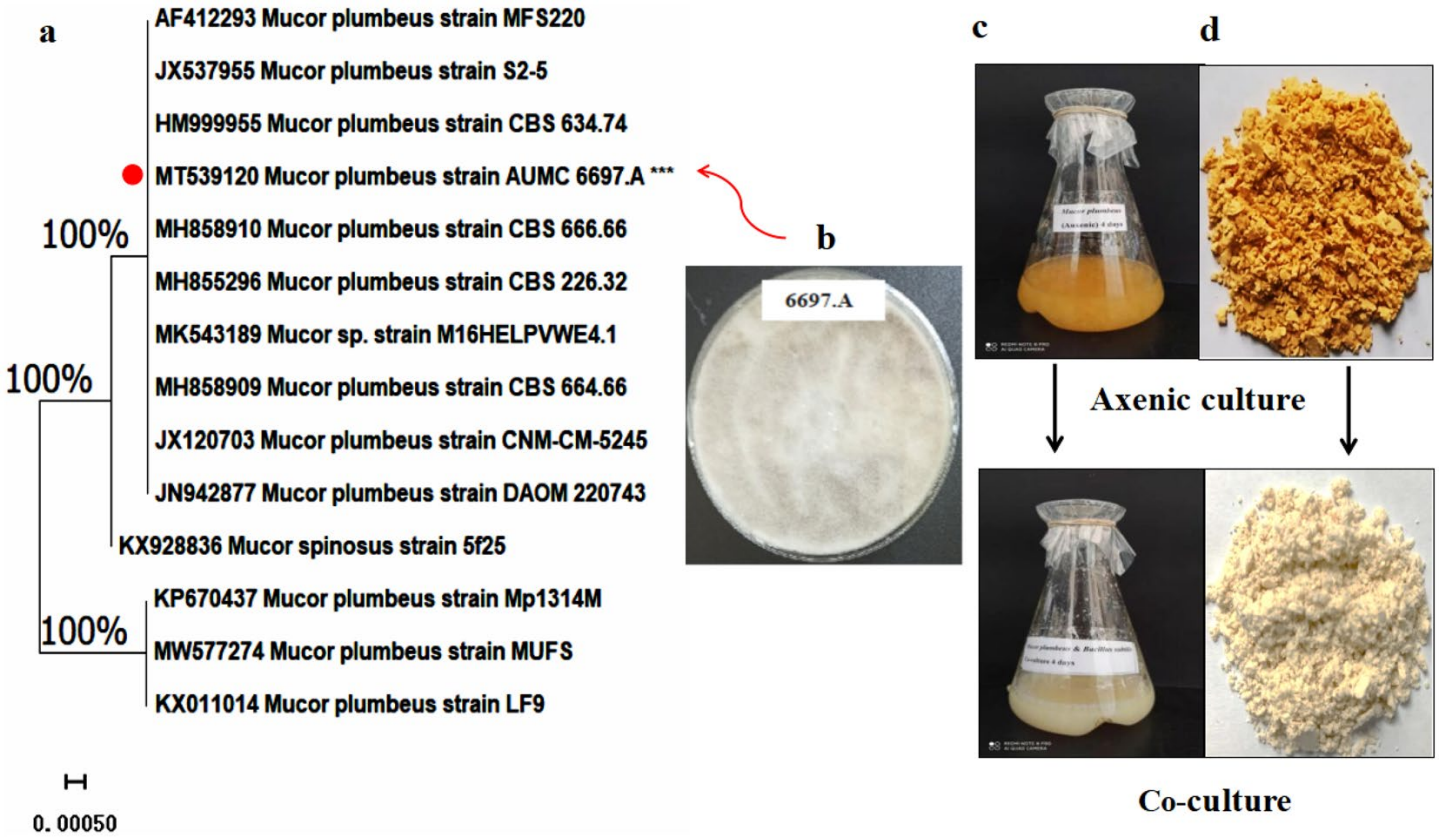
(**a**) The neighbour-joining (NJ) phylogenetic tree based on ITS gene sequences for AUMC 6697.A with closely related strains in the GenBank database aligned using ClustalW. Bootstrap values included 500 replicates of the NJ method using the MEGA software (v.11.0.6). *** denotes the target strain. (**b**) Culture characteristics. (**c**) Growth behaviour and colouration of 6697.A in axenic cultures and cocultures after 4 days of fermentation. (**d**) Lyophilised biomass of 6697.A in axenic cultures and cocultures after 4 days of fermentation.

Based on the maximum produced GLA yield obtained with *M. plumbeus* 6697.A during our initial coculturing, ~ twofold higher than its monoculture, we selected this fungal strain for further analysis. The target isolate was described as fast-growing Mucormycota that creates colonies that are initially pale white but eventually become dark grey on solid potato dextrose agar (PDA) plates, with unicellular coenocytic sporangiophore carrying dark rounded sporangium and sporangiospores. The results of internal transcribed spacer (ITS) ribosomal RNA (rRNA) gene sequences of *M. plumbeus* were clustered with similar species, consistent with previous phylogenetic reconstructions (Fig. 2).

**Figure 3:**
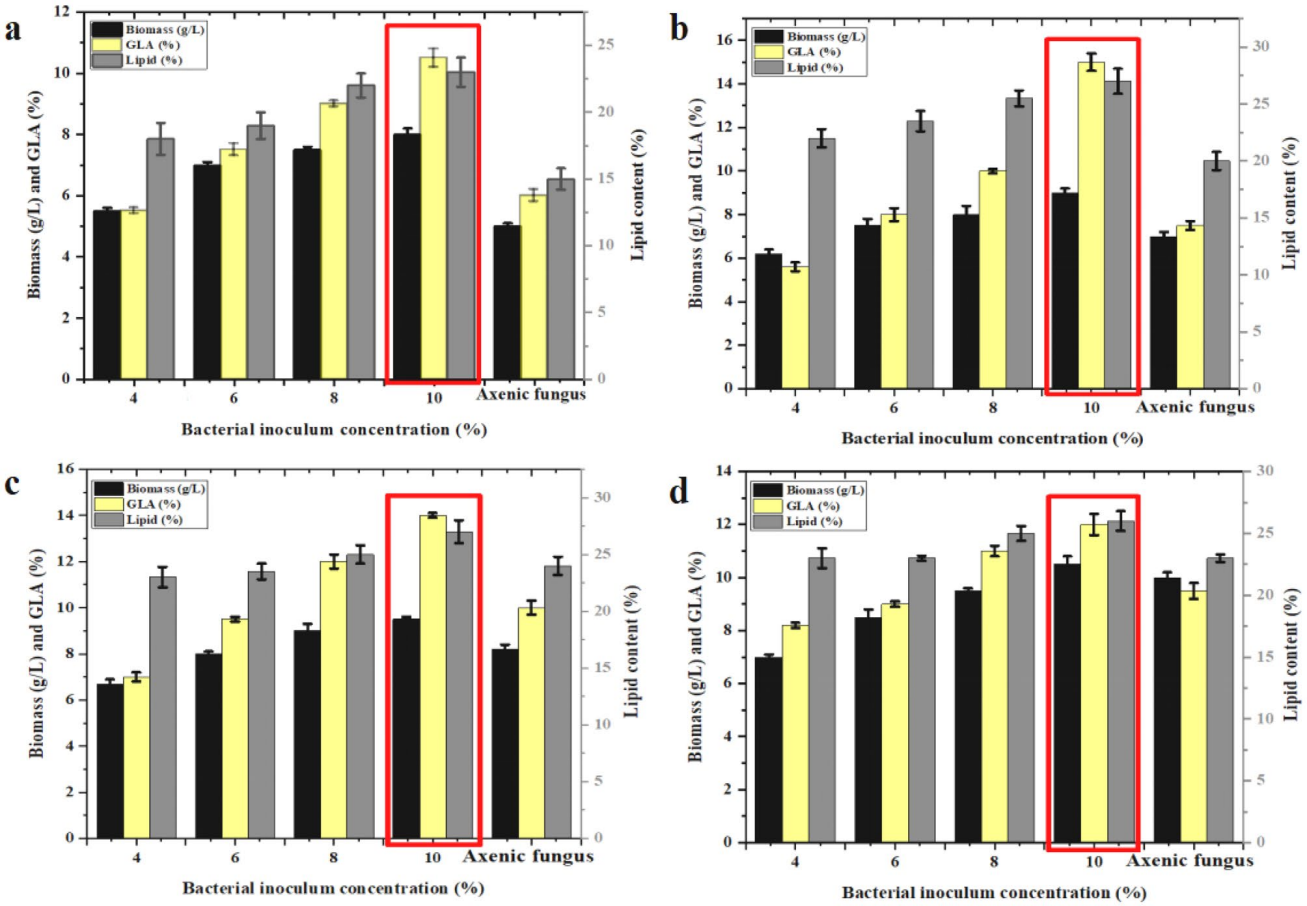
Effect of different bacterial inoculum concentrations cocultured in K&R fermentation media with target fungal strain 6697.A on biomass, lipid, and GLA yields at (**a**) 24 h, (**b**) 48 h, (**c**) 72 h, and (**d**) 96 h. Error bars represent the SD across three biological replicates.

Four different concentrations of *B. subtilis* (4%, 6%, 8%, and 10% [*v*/*v*]) were assessed with *M. plumbeus* added at different cocultivation fermentation periods under aseptic conditions as fed-batch fermentation (24, 48, 72, and 96 h). The results showed that the biomass, lipid, and GLA yields reached their maximum levels at 10% (*v*/*v*) of bacterial inoculum for all coculturing periods, increasing with bacterial inoculum concentration. In particular, biomass, lipids, and GLA yields reached 9.1 ± 0.2 g/L, 27.2 ± 1.2%, and 15 ± 0.4% of TFAs, respectively, after 48 h of coculture fermentation, their highest values across all cocultivation periods (Fig. 3). Moreover, the lowest biomass, total lipid, and GLA yields were seen after 24 h of coculturing at all concentrations of *B. subtilis*. It was observed that the 12–20% of bacterial inoculum concentrations were also investigated and showed less effective as compared to 10%. The reason may be the bacteria load in high inoculum grows very high that may exhaust the media too earlier. Too low of bacterial inoculum is not sufficient to elicit the fungal lipid production.

### Influence of different carbon sources

The influences of several carbon sources (galactose, glucose, fructose, xylose, sorbitol, cellobiose, glycerol, and sucrose) on dry biomass, lipid, and GLA yields from coculture fermentation of *M. plumbeus* and *B. subtilis* are shown in Table 1. The cocultures can utilise each of the monosaccharides (glucose, galactose, xylose, and fructose), disaccharides (cellobiose and sucrose), and sugar alcohols (sorbitol and glycerol). Most of these carbon sources significantly increased lipid and GLA yields, ranging from 15.5 ± 0.2 to 32 ± 1.15% and 7.69 ± 0.1 to 25.06 ± 0.3% of dry cell biomass, respectively. Coculture fermentation produced maximum lipid yields but the lowest GLA yields with galactose, fructose, and xylose. Moreover, the highest GLA yields were obtained with sorbitol, cellobiose, sucrose, and glycerol. Notably, all carbon sources assessed produced higher biomass quantities except glucose, glycerol and sucrose. Using galactose as the sole carbon source in the coculture fermentation medium provided a volumetric biomass production rate (QX) of 2.86 ± 0.02 (g/L/d) with a lipid yield of 32 ± 1.15%, and GLA yield was highest with cellobiose, sucrose, and glycerol as a sole energy source (19.89 ± 0.2, 25.06 ± 0.47, and 22.61 ± 0.28%, respectively). In addition, the lowest yields of dry biomass and total lipid content were with sucrose. Therefore, the highest biomass, lipid, and GLA yields were all observed when cellobiose was the sole carbon source, which was selected for the next experiment.

**Table 1:** Influence of different carbon sources on dry biomass, GLA, and lipid production parameters by coculture fermentation.

Parameter	Tested carbon sources								
	Glucose	Galactose	Fructose	Xylose	Sorbitol	Cellobiose	Sucrose	Glycerol	Axenic fungus (control)*
Dry biomass (g/L)	8.76 ± 0.3	11.44 ± 0.4	11.10 ± 0.2	10.81 ± 0.1	10.50 ± 0.16	10.80 ± 0.12	8.20 ± 0.15	9.77 ± 0.12	10.3 ± 0.34
Lipid yield (g/L)	1.79 ± 0.01	3.66 ± 0.06	2.99 ± 0.02	2.97 ± 0.01	2.25 ± 0.01	2.52 ± 0.02	1.27 ± 0.01	2.34 ± 0.03	2.28 ± 0.02
Specific yield of lipid (Yp/x)	0.20 ± 0.01	0.32 ± 0.01	0.27 ± 0.03	0.27 ± 0.02	0.21 ± 0.01	0.24 ± 0.02	0.15 ± 0.01	0.24 ± 0.01	0.22 ± 0.02
Lipid content (%)	20.5 ± 0.6	32 ± 1.15	27 ± 0.14	27.5 ± 0.35	21.5 ± 0.32	25 ± 0.50	15.5 ± 0.2	24 ± 0.25	22.1 ± 1.22
GLA content (%,TFA)	10.03 ± 0.1	7.69 ± 0.1	8.05 ± 0.1	9.34 ± 0.3	16.89 ± 0.3	19.89 ± 0.2	25.06 ± 0.4	22.61 ± 0.28	8.1 ± 0.1
Volumetric biomass production rate,Qx (g/L/d)	2.19 ± 0.06	2.86 ± 0.02	2.77 ± 0.03	2.70 ± 0.02	2.62 ± 0.03	2.53 ± 0.02	2.05 ± 0.01	2.44 ± 0.02	2.58 ± 0.04
Volumetric lipid production rate (Qp)	0.44 ± 0.00	0.91 ± 0.01	0.74 ± 0.01	0.74 ± 0.02	0.56 ± 0.00	0.60 ± 0.01	0.31 ± 0.00	0.58 ± 0.01	0.57 ± 0.01

*Axenic fungus cultured on K&R medium containing glucose as a carbon source and ammonium tartrate as a nitrogen source at a pH 6.0 and 28 °C for 4 days.

### Influence of cellobiose concentration

**Figure 4:**
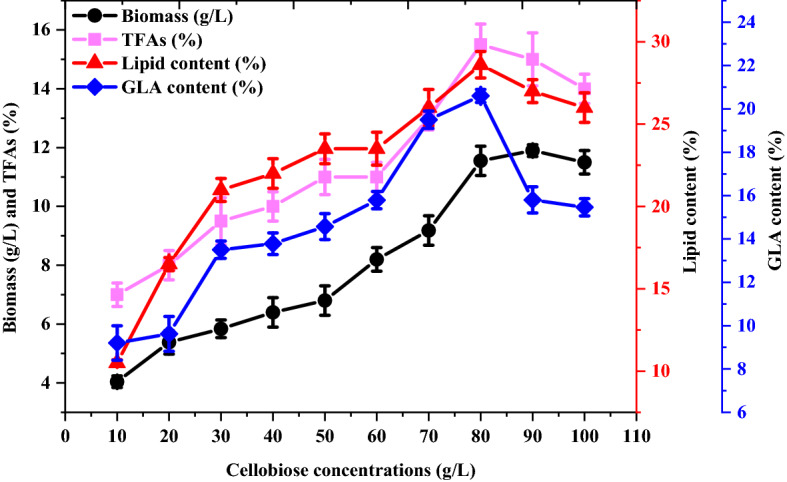
Influence of various cellobiose concentrations as the carbon source on biomass (g/L), lipid (%), TFA and GLA (%) yields from *M. plumbeus* and *B. subtilis* cocultures*.*

Ten different concentrations of cellobiose were used to determine the coculture fermentation concentration at which lipid and GLA yields were greatest. Obtained biomass, lipid, and GLA yields gradually increased with cellobiose concentrations between 10 and 100 g/L (Fig. 4). While the highest lipid (28 ± 1.2) and GLA (20.5 ± 0.8%) yields were achieved with 80 g/L of cellobiose in the culture media, the highest fungal biomass yield (11 g/L) was achieved with 90 g/L of cellobiose. Increasing the primary cellobiose concentration above 80 g/L led to changes in the total lipid and GLA yields but no significant increase in fungal biomass.

### Influence of different nitrogen sources

We next explored the influences of different nitrogen sources on cocultures of *M. plumbeus* and *B. subtilis*. Their capability to use different organic (ammonium tartrate, yeast and malt extracts, peptone, casein, and urea) and inorganic (sodium nitrate, ammonium chloride, and ammonium sulphate) nitrogen sources in the fermentation medium to enhance the biomass, lipid, and GLA yields is shown in Table 2. Maximum lipid and GLA yields were achieved with organic nitrogen sources, ranging from 1.48 ± 0.01 to 2.83 ± 0.01 g/L and 11.53 ± 0.02 to 16.23 ± 0.02% of TFA, respectively. The use of inorganic nitrogen sources provided moderate to high lipid yields (2.44 ± 0.01 to 3.62 ± 0.03 g/L), but GLA yield varied from 10.71 ± 0.1 to 16.23 ± 0.2% of TFA. Our results indicate that ammonium sulphate is the optimal nitrogen source based on biomass, lipid, and GLA yields during coculturing of *M. plumbeus* and *B. subtilis*, with a total lipid yield of 28 ± 0.33% and volumetric biomass production rate of 2.23 ± 0.01 (g/L/d). The use of organic nitrogen sources in cocultures provides low lipid and dry biomass yields, with the lowest yields obtained with urea (14.5 ± 0.01% and 10.24 ± 0.01 g/L, respectively).

**Table 2:** Influence of different nitrogen sources on dry biomass, GLA and lipid production parameters by coculture fermentation.

Parameter	Organic nitrogen sources						In-organic nitrogen sources			
	Ammonium tartrate	Yeast extract	Malt extract	Peptone	Casein	Urea	Sodium nitrate	Ammonium chloride	Ammonium sulfate	Axenic fungus (control)*
Dry biomass (g/L)	10.5 ± 0.2	9.14 ± 0.1	9.12 ± 0.1	10.88 ± 0.2	9.66 ± 0.1	10.24 ± 0.1	8.93 ± 0.1	10.88 ± 0.2	12.95 ± 0.11	10.4 ± 0.22
Lipid yield (g/L)	2.83 ± 0.01	1.91 ± 0.01	2.69 ± 0.01	2.72 ± 0.01	2.51 ± 0.02	1.48 ± 0.01	2.50 ± 0.02	2.44 ± 0.01	3.62 ± 0.03	2.34 ± 0.04
Specific yield of lipid (Yp/x)	0.27 ± 0.0	0.21 ± 0.01	0.29 ± 0.0	0.25 ± 0.01	0.26 ± 0.02	0.14 ± 0.0	0.28 ± 0.02	0.22 ± 0.0	0.28 ± 0.02	0.22 ± 0.03
Lipid content (%)	27 ± 0.35	21 ± 0.18	29.5 ± 0.50	25 ± 0.22	26 ± 0.38	14.5 ± 0.10	28 ± 0.25	22.5 ± 0.35	28 ± 0.33	22.5 ± 1.30
GLA content (%, TFA)	13.7 ± 0.1	11.54 ± 0.3	11.53 ± 0.2	12.43 ± 0.3	11.89 ± 0.2	16.23 ± 0.2	11.15 ± 0.1	10.71 ± 0.1	13.39 ± 0.1	9.5 ± 0.1
Volumetric biomass production rate,Qx (g/L/d)	2.62 ± 0.01	2.28 ± 0.01	2.28 ± 0.01	2.72 ± 0.02	2.41 ± 0.01	2.56 ± 0.02	2.23 ± 0.02	2.72 ± 0.02	2.23 ± 0.01	2.6 ± 0.05
Volumetric lipid production rate (Qp)	0.70 ± 0.01	0.47 ± 0.0	0.67 ± 0.01	0.68 ± 0.02	0.62 ± 0.01	0.37 ± 0.0	0.62 ± 0.01	0.61 ± 0.01	0.90 ± 0.03	0.59 ± 0.02

*Axenic fungus cultured on K&R medium containing glucose as a carbon source besides ammonium tartrate and yeast extract as nitrogen sources at pH 6.0 and 28 °C for 4 days.

### Influence of ammonium sulfate concentration

**Figure 5:**
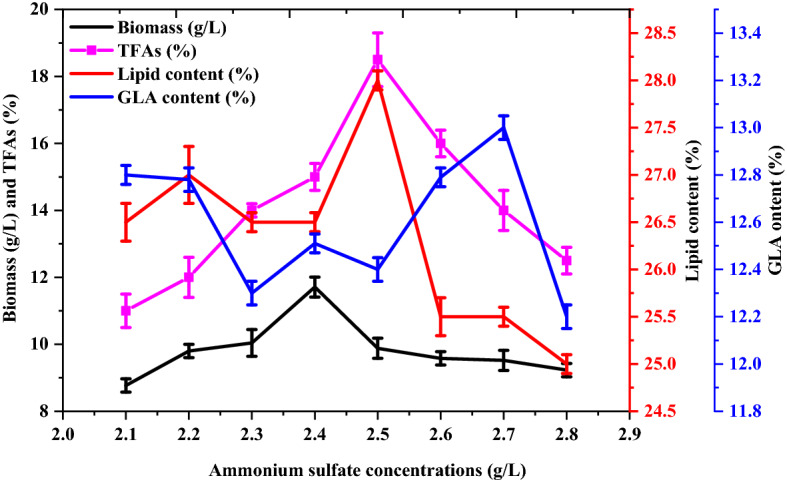
Effect of different ammonium sulfate concentrations as the nitrogen source on biomass (g/L), lipid (%), TFA and GLA (%) yields from *M. plumbeus* and *B. subtilis* cocultures*.*

Based on maximum biomass, lipid, and GLA yields obtained with ammonium sulfate, it was selected as the inorganic nitrogen source for further analysis. The effects of different concentrations of ammonium sulfate (2.1–2.8 g/L) as the sole nitrogen source on dry biomass, lipid, and GLA yields in cocultures of *M. plumbeus* and *B. subtilis* are shown in Fig. 5. Maximum biomass yield (12 ± 0.02 g/L) was obtained with 2.4 g/L of ammonium sulfate, and maximum lipid (28 ± 0.9) and GLA (13 ± 0.2%) yields were obtained with 2.5 and 2.7 g/L of ammonium sulfate, respectively.

### Influence of environmental factors during coculturing

**Figure 6:**
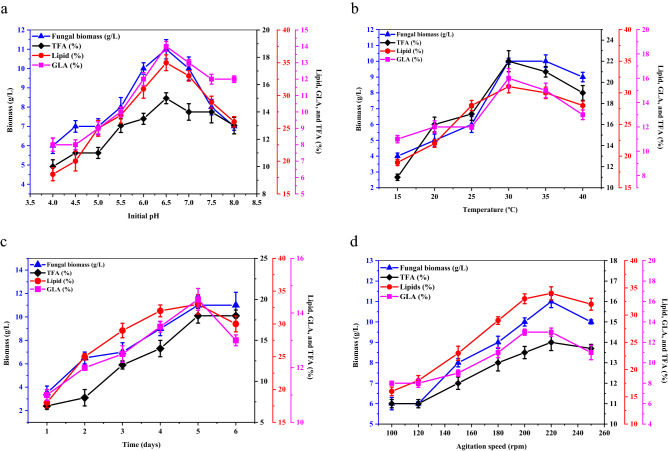
Effect of fermentation time, temperature, initial pH, and agitation speed on the biomass, total lipid, GLA, and TFA yields in *M. plumbeus* and *B. subtilis* cocultures. Error bars represent the SD across three biological replicates.

K&R fermentation media containing cellobiose and ammonium sulfate as the sole carbon and nitrogen sources, respectively, was used to explore the effects of environmental factors such as fermentation time, pH, temperature, and agitation speed on the production of biomass, total lipids, TFA, and GLA in cocultures of *M. plumbeus* and *B. subtilis* incubated for 1 to 6 days (Fig. 6). The highest yields of GLA, as a proportion of TFAs (14.5 ± 0.4% of 18.2 ± 0.6%), total lipids, as a percentage of CDW (33 ± 1.5%), and biomass (11.2 ± 0.7 g/L) were obtained after five days of coculturing.

Cocultures with fermentation media at pH between 4 and 8 incubated at 28 °C for 4 days were assessed, and the greatest yields of total lipids (35 ± 1.2% of CDW) and biomass (11 ± 0.2 g/L) were obtained at pH 6.5. Notably, total biomass and lipid yields dropped significantly at sub-optimal pH. However, the maximum yields of TFA (15 ± 0.4%) and GLA (14 ± 0.2%) were also obtained at pH 6.5. Cocultures were inoculated in fermentation media and incubated at temperatures between 15 and 40 °C for 4 days to explore the effects of temperature. Biomass yields were greatest between 30 and 35 °C (10 ± 0.2 g/L), while total lipid (31 ± 0.8), TFA (22 ± 0.4), and GLA (6 ± 0.2%) yields were greatest at 30 °C. Finally, total lipid (34 ± 1.2%), biomass (11 ± 0.3 g/L), and TFA (14 ± 0.4%) yields were greatest at an agitation speed of 220 rpm, while the GLA yield (13 ± 0.1%) was greatest at 200–220 rpm.

### Scale-up of cocultures in a stirrer bioreactor

**Figure 7:**
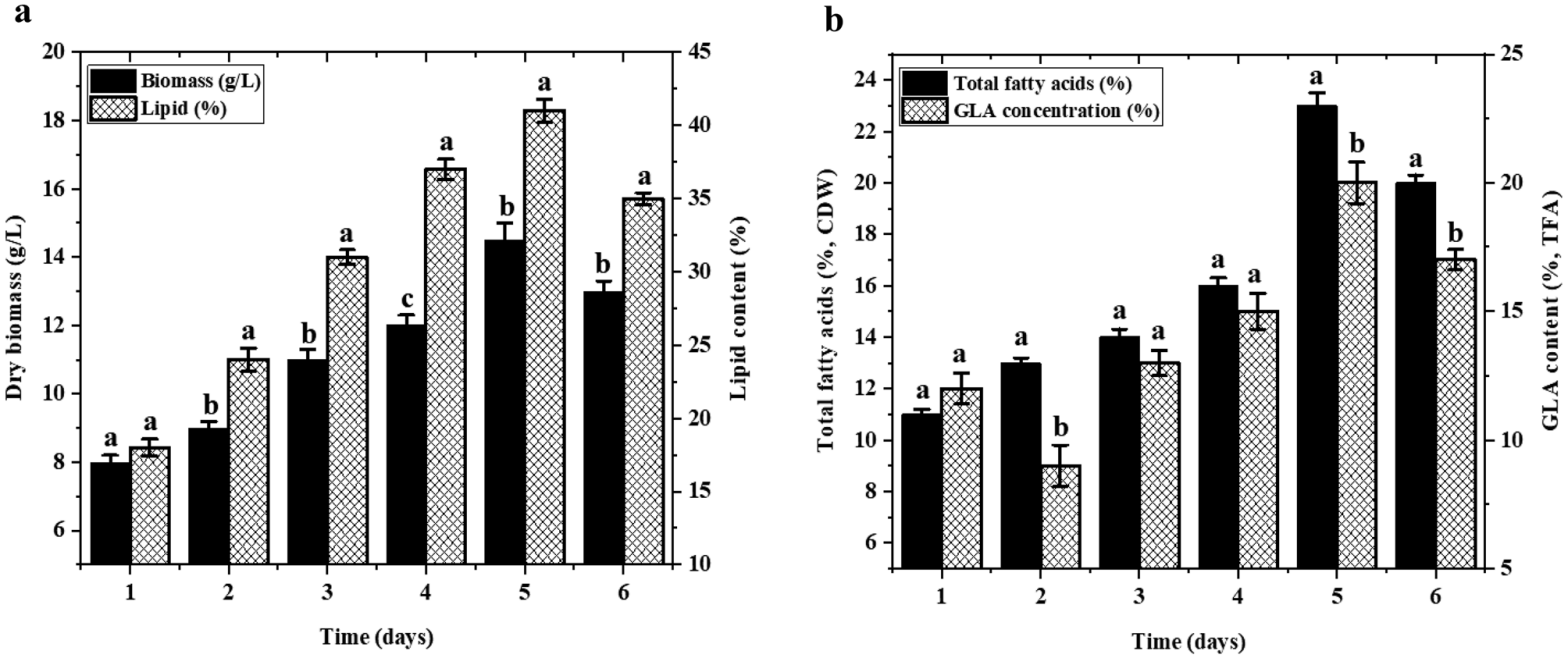
(**a**) Growth and lipid, and (**b**) TFAs and GLA yields (%) of *M. plumbeus* and *B. subtilis* cocultures incubated in a 5 L fermenter containing K&R fermentation media at 30 °C, agitation at 250 rpm, and aeration at 1 vvm min^−1^ for 6 days.

We next scaled up our cocultures using a 5 L bench-top fermentor with 3 L working volumes to investigate the reproducibility of the optimal nutritional and environmental conditions determined above in large-scale production. Cocultures were found to produce up to 14.5 ± 0.4 g/L biomass, 41.5 ± 1.3% total lipids, 24 ± 0.8% TFAs, and 20 ± 0.5% GLA (Fig. 7), significantly more than the yields obtained in shaker flasks under the same conditions (Fig. 1). Potential explanations for this increase include better oxygen supply and agitation in a larger space, improving growth and GLA production. Consequently, these findings demonstrate the reproducibility and applicability of shaker flasks at a 5 L scale and their utility for assessing coculturing strategies for large-scale industrial production. The chemical investigation of cocultures also showed multiple molecules that are soluble in non polar solvents (total lipids) including carotenoids, sterol, glycolipids, and lipoprotein in various concentrations. However, we focused only on total fatty acids due their importance on potential biodiesel properties and the presence of PUFAs using specific GC protocols.

### Production and characterisation of FAs

**Figure 8:**
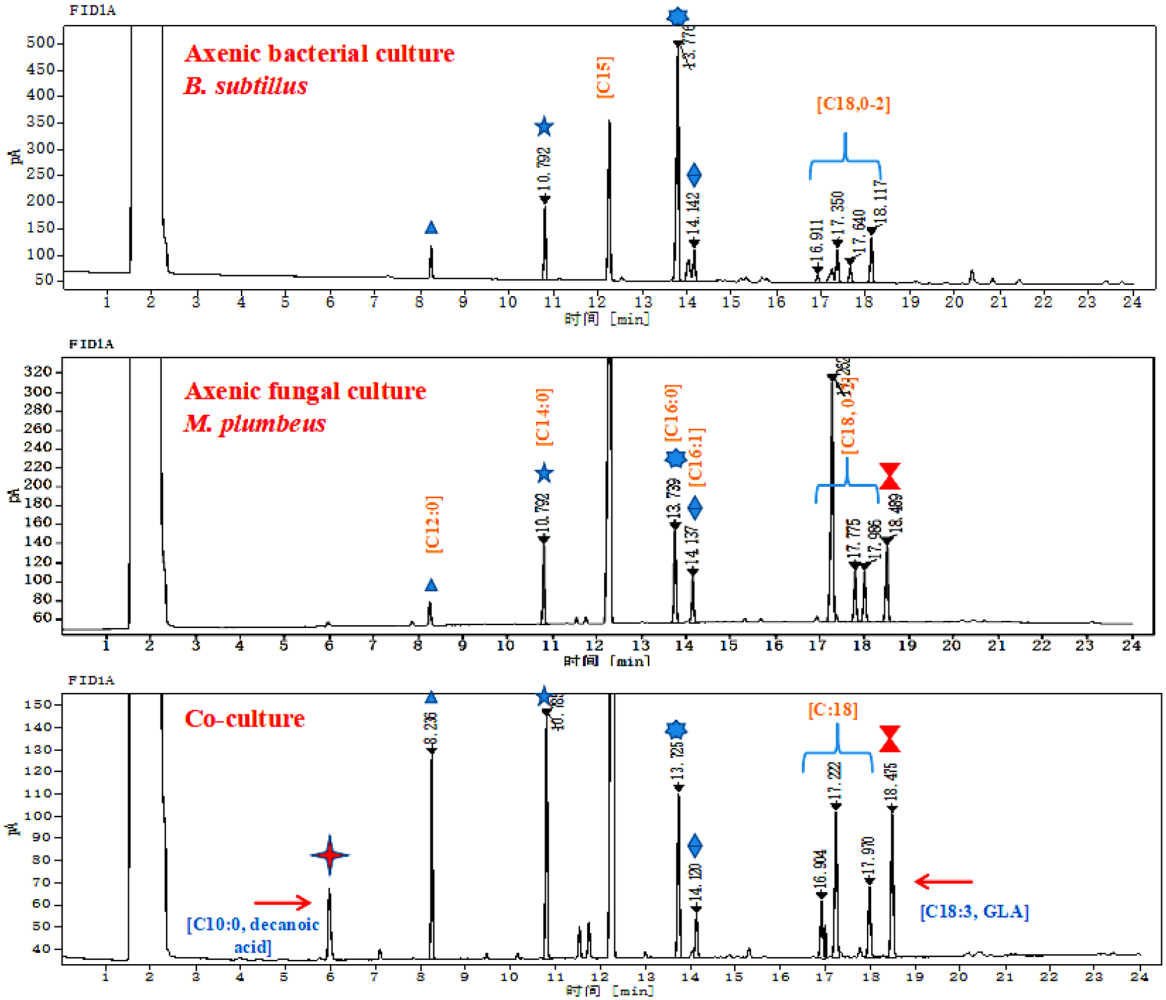
The GC chromatograms of FAs from axenic and cocultures of *M. plumbeus* and *B. subtilis* after 6 days of fermentation. Note that C10:0 is an induced FA.

We explored the effect of coculture fermentation on biomass, lipid, and FA yields by *M. plumbeus* and *B. subtilis.* The FA profiles of cocultures and monocultures primarily encompassed 16–18 carbon (C) atoms (Table 3 and Fig. 8). Our findings show that the content of C12 and C14 in cocultures reaches 17.25 ± 1.15% and 20.26 ± 0.11%, respectively, significantly higher than their production in bacterial and fungal monocultures. A significant decrease in C18:1 yield was observed in cocultures (14.16 ± 1.15%) compared to bacterial and fungal monocultures, with C16:1 the FA with the lowest abundance (3.17 ± 0.01%). Interestingly, the GLA yield of cocultures was significantly increased (18.10 ± 0.49%), more than twice that of fungal monocultures (8.27 ± 0.11%); bacteria do not produce GLA. More interestingly, GC chromatograms for cocultures show the induction of C10:0 (capric acid; also called decanoic acid), along with other well-known *M. plumbeus* FAs, with a yield of 5.32 ± 0.20%, which is not detected in bacterial or fungal monocultures (Table 3 and Fig. 8). The coculturing results indicate that high GLA levels and C10:0 production may directly affect lipid yields, and coculture fermentation may be an efficient strategy for lipid and oil production for various industrial applications.

**Table 3:** Fatty acids profiles through *Mucor plumbeus* and *Bacillus subtilis* cocultures.

Fatty acid(s)	Lipid number	Composition of fatty acids (% TFA)			Molecular formula
		Axenic bacteria	Axenic fungus	Co-culture (5 L fermentor)	
Capric acid*	C10:0	ND	ND	5.32 ± 0.20	C_10_H_20_O_2_
Lauric acid	C12:0	6.80 ± 0.04	3.91 ± 0.20	17.25 ± 1.15	C_12_H_24_O_2_
Myristic acid	C14:0	11.95 ± 0.80	9.58 ± 0.11	20.26 ± 0.11	C_14_H_28_O_2_
Palmitic acid	C16:0	49.66 ± 1.20	17.69 ± 0.40	15.13 ± 0.33	C_16_H_32_O_2_
Palmitoleic acid	C16:1	12.39 ± 1.10	4.03 ± 0.75	3.17 ± 0.01	C_16_H_30_O_2_
Stearic acid	C18:0	3.61 ± 0.01	4.46 ± 0.08	3.26 ± 0.06	C_18_H_36_O_2_
Oleic acid	C18:1	11.44 ± 0.30	39.48 ± 1.21	14.16 ± 1.15	C_18_H_34_O_2_
Linoleic acid	C18:2	9.54 ± 0.08	13.49 ± 1.25	5.22 ± 0.65	C_18_H_32_O_2_
Gamma linolenic acid	C18:3	ND	8.27 ± 0.11	18.10 ± 0.49	C_18_H_30_O_2_

“ND”: not detected.

*New peak was induced during co-cultivation.

## Discussion

Oleaginous microbes, including fungi, are considered an alternative source for lipid derivatives, particularly PUFAs. Many important fungal lipids have been studied using different approaches such as metabolic engineering, high-throughput screening, genome mining, and leveraging new microbial resources^24,25^. While lipid production by several oleaginous candidates has been extensively studied, the coculture method has not been explored for bacterial-fungal derived lipids. However, while it has shown its versatility in algal studies focusing on various types of nutritional growth^17^, its utility for producing important products such as lipids in the context of bacterial and fungal symbiotic relationships remains to be explored.

In this study, eleven Mucormycota were cocultured with *B. subtilis* to explore the potential of their coculturing for increasing biomass, total lipid, FA, and GLA yields. Our preliminary results show that the productivity of most of the tested fungi significantly improved. In particular, *M. plumbeus* strain 6697.A was selected for further analysis based on its high GLA yield, which was twofold higher than in its monocultures. Therefore, cocultures represent an efficient strategy that can be exploited for lipid and biomass production. Coculturing fungi and bacteria better emulates their natural living environment and may enable the activation of silent genes or gene clusters in both organisms through interspecies interactions^26^. The coculturing of different microorganisms often improves their production of various compounds and can lead to the production of cryptic compounds not detected in monocultures under various culturing conditions^27^.

Recently, coculturing was broadly used in exploring unexplored biosynthetic potentials, such as new enzymes and metabolites, that may be employed in various fields in the future, including agriculture, medicine, and industry^28^. For example, enhancing FA composition in the soybean fermentation process through coculturing with *B. subtilis* and *R. oligosporus*^29^. Moreover, the coculturing of *Heveochlorella* sp. and *Monoraphidium* sp. FXY-10 improved the efficiency of lipid productivity and flocculation to 70.55% and 203.8 mg L^−1^ day^−1^, respectively, compared to monocultures^10^. Another study reported that *C. vulgaris* growth was significantly enhanced in the presence of *A. brasilense*, with lipid yields increasing appreciably by 6.76–32.94% under monoculture and coculture conditions, respectively^30^. Moreover, it was reported that the biomass and lipids yields of microalga *E. gracilis* increased by almost 3.2-fold and 2.9-fold, respectively, when cocultured in a medium of non-sterilised wastewater effluent containing *Emticicia* sp. EG3 bacteria^19^. Further studies are required to evaluate whether the beneficial effects of coculturing found by this study on lipid and FA yields are a consequence of changes in important gene regulation or basic metabolite influences.

The ITS region is an important fast-evolving constituent in the rRNA cistron and an effective marker for fungal identification. Many studies have used the ITS as a convenient DNA barcode for fungal systematics and taxonomy to investigate relationships of various species based on large barcoding gaps^31^. Consequently, the ITS alone or combined with protein-coding genes may be appropriate for identifying and classifying fungal strains^32^. For example, the ITS sequencing fragment for strain 6697.A showed excellent congruence with high sequence similarities (100%) across all correlated strains in the phylogenetic tree for *M. plumbeus.*

Inoculum size plays an essential role in cell metabolism, and our findings indicate that 10% (*v/v*) was the optimal bacteria inoculum size for our coculturing system. Similarly, coculturing of the yeast *Trichosporonoides spathulata* with microalga *C. vulgaris* var. *vulgaris* TISTR 8261 increased total dry biomass and lipid yields by nearly 1.6-fold over yeast monocultures after 2 days and by 1.1-fold after 3 days^33^. A recent study found the bacterium *B. subtilis* strain BS-15 to increase the plantaricin yields of *Lactiplantibacillus plantarum* strain RX-8 in cocultures with a 1:1 inoculum ratio (10^6^:10^6^ CFU/ml of cell suspension) compared to *L. plantarum* RX-8 monocultures^34^.

Overall, several studies have shown that nutritional factors, including carbon and nitrogen sources, play an important role in lipid and FA production in several oleaginous microorganisms^35,36^. In addition, the broad adaption of fungal strains to multiple carbon and nitrogen sources enables them to consume different biomaterials as substrates^37^. For example, when pure glycerol (3%) and urea were used as carbon and nitrogen sources, respectively, in cocultures of oleaginous yeast *R. glutinis* and microalga *C. vulgaris*, total lipid and cellular biomass yields were up to 3.8-fold and 5.7-fold higher, respectively, compared to the initial monocultures^38^.

In an earlier study, the optimal carbon source for *M. alpina* strain TSM-3 was cornflour when yeast extract was used as the nitrogen source, producing ARA (31.77% of TFA, 3.58 g/L). While *M. alpina* strain SL-4 produced maximum lipid (4.34 g/L) and ARA (1000 mg/L) yields with glucose as the carbon source, it produced 4.64 g/L of lipids and 950 mg/L of ARA with yeast extract as the nitrogen source^37^. Given the importance of the carbon source, a high C/N ratio of 120 produced up to 60% more lipids with xylose but lower biomass. In addition, culturing of *R. toruloides* with glycerol yielded the most biomass and total lipids (0.085 g) at C/N ratios of 60 and 100^39^. Similar to our study, Rasmey et al. explored various carbon and nitrogen sources with *Fusarium solani* RAS18 to optimise its biomass, lipids, and fatty acid production, obtaining the highest lipid yield (34.5%) with peptone (35 g/L) and glycerol (35 g/L) as the nitrogen and carbon sources, respectively^40^. In a previous coculturing study between *Lipomyces starkeyi* yeast and *Bacillus cereus* bacterium for lipid accumulation capability using palm oil mill effluent (POME) as a carbon source, provided 2.95 g/L of total lipids where inoculum composition, pH, temperature, and incubation time reported 50:50, 6.50, 32.5 °C, and 90 h, respectively^41^. Many other species have been reported to have high growth coupled with other microbial species, such *R. glutinis* and *Ambrosiozyma cicatricose*^42–44^ and *Monoraphidium* sp FXY10^45,46^. The composition of the coculture inoculum can have a fundamental effect on the performance of microbial growth and lipid production in a coculture system. Furthermore, numerous environmental conditions, such as pH, incubation time, and varied temperature, can significantly affect the production of lipids of microbial origin^47,48^. According to a recent study, the total lipid and FAs profiles vary depending on the behaviour of microbes and abiotic variables such as nutritional substrate variety and its quantities, cultivation temperature, incubation time, initial pH, nutrients, and standing and agitation conditions^49^. Consequently, optimising operational parameters is becoming more important for improving the efficiency of the microbial culturing process and lipid yields^48^. The lipid and GLA yields produced by multiple Mucorales members, such as *M. rouxii* and *M. circinelloides*, are well known. For example, using the central composite rotatable design (CCRD) in combination with the statistical response surface methodology (RSM) to identify optimal conditions, the GLA yields of the oleaginous fungus *M. rouxii* strain CFR-G15 reached up to 18.55%^50^. Similarly, GLA yields increased when *U. isabellina* was cultured in broth containing 1% yeast extract and 2% octadecanol^51^, demonstrating the significant potential of oleaginous fungi in PUFA production under optimal culturing conditions.

Moreover, C16:0 and C18:1 FAs were produced in large quantities, indicating that the coculture strategy of *A. awamori* and *C. minutissima* strains MCC 27 and UTEX 2219 is an efficient and promising approach for lipid and oil production, reaching yields up to 31.26–35.02% and 21.14–24.21%, respectively^30^.

The results of this study not only indicate that coculturing can improve GLA yield ~ twofold but that it also induces the production of a new FA (decanoic acid), highlighting the importance of comparing the chemical composition of cocultures with their respective monocultures (Fig. 8). Altogether, microbial cocultures such as fungus-bacterium show that they interact mutually through metabolites exchange. These cocultures provide the opportunity to convert to renewable sources for bioproducts, enhance yield and production, improve substrate utilisation rate, and reduce processing costs^13^. However, the application of microbial cocultures for large-scale lipid and oil production in industrial fermentation still poses many challenges, such as the molecular mechanism of lipid production and signalling pathways and the stability of microbial species under coculturing. Further studies are required to robustly overcome these difficulties and provide lipid and FA biosynthesis in high quantities.

## Conclusions

This study improved the production of total lipids and Fats, including GLA, produced by fungus *M. plumbeus* strain 6697.A cocultured with bacterium *B. subtilis* using cellobiose as a carbon source and explored the influences of nutritional and abiotic factors. Coculturing significantly increased yields of fungal biomass, lipids, total FAs, and GLA, which doubled. Additionally, decanoic acid was produced, which was not detected in bacterial or fungal monocultures. Further study of coculture fermentation strategies is essential for understanding the molecular mechanism of lipid production in the context of fungal-bacterial interactions.

## Materials and methods

### Microbial cultures

This study used 11 *Mucoromycota* fungal strains isolated in our previous work^2^ (Supplementary Table S1). *Bacillus subtilis* bacterial strain ATCC 6633 was obtained from the Bacteriology Laboratory et al.-Azhar University, Egypt. For inoculum preparation, the fungal strains were cultured and maintained in a PDA medium containing 200 g/L potato infusion, 20 g/L dextrose, and 20 g/L agar in 1 L distilled water with a final pH of 5.6 ± 0.2 at 28 °C for 3‒4 days. The fungal spores (10^6^ spores/mL) were then harvested and resuspended in a sterile 0.85% saline solution. The bacterium was grown in a nutrient broth (NB) medium containing 3 g/L beef extract, 5 g/L peptone, and 5 g/L NaCl in 1 L distilled water with a final pH of 6.8 ± 0.2 at 37 °C for 24 h. The cells (10^4^ cells/mL) were then harvested and resuspended in a sterile 0.85% saline solution.

### Coculture fermentation conditions

The K&R fermentation medium of the selected *Mucoromycota* members was used to improve lipid production. 0.1 mL of each fungal spore suspension (10^6^–10^7^ spores/mL) was first transferred to 500 mL baffled flasks containing 150 mL K&R medium^52^ containing 3.3 g/L ammonium tartrate, 30 g/L glucose, 2 g/L disodium phosphate, 7 g/L monopotassium phosphate, 1.5 g/L yeast extract, 1.5 g/L magnesium sulphate, 0.1 g/L calcium chloride, and a mineral stock solution containing 0.1 mg/L manganese sulphate, 8 mg/L iron chloride, 0.1 mg/L copper sulphate, 1 mg/L zinc sulphate, and 0.1 mg/L cobalt nitrate dissolved in 1 L distilled water according to Naz et al.^53^. All media were autoclaved for 20 min at 121 °C before use. Flasks were then incubated at 30 °C for 24 h. with an agitation speed of 150 rpm before the addition of 10% (*v*/*v*) inoculate to 1 L baffled flasks containing the same fermentation media composition (150 mL) except for glucose and ammonium tartrate, which were at 80 g/L and 2 g/L, respectively. The co-fermentation flasks were then prepared using 15 mL of bacterial inocula (10^4^ CFU) as the inoculum for coculturing (11 flasks) before incubation at 30 °C for 96 h with an agitation speed of 150 rpm. Based on the strain producing the highest lipid and GLA yields during coculturing, four inoculations at 24, 48, 72, and 96 h with different concentrations of *B. subtilis* (4%, 6%, 8%, and 10% (*v*/*v*)) were used, in addition to one flask of axenic *B. subtilis* and one flask of axenic fungal strain. Finally, all culturing flasks were incubated for 96 h at 30 °C.

### Cell dry weight (CDW) determination

The culture mycelium was harvested and filtered as described previously by Nosheen et al.^52^. In brief, the obtained biomass was washed thrice with distilled water during filtration steps using a Buchner funnel to remove the medium before being frozen at -80 °C for 15 h and lyophilised (freeze-dried) for 2 days. The obtained biomass was reported as g/L of fermentation medium.

### Effect of nutritional conditions

#### Effect of different carbon sources

Eight carbon sources (galactose, glucose, fructose, xylose, sorbitol, cellobiose, glycerol, and sucrose) were used in the coculturing medium to determine their effects on lipid and GLA production. These carbon substrates were tested individually to reach a mass concentration of 80 g/L and added along with the other K&R fermentation medium components. Coculturing flasks were adjusted to pH 5.5 with 0.1 M sodium hydroxide and hydrochloric acid. The coculturing fermentation medium then was inoculated with *M. plumbeus* and *B. subtilis* (10% seed culture) and incubated at 28 °C for 4 days with agitation at 150 rpm. The biomass was then harvested and used for CDW and lipid analyses.

#### Effect of cellobiose concentration

Different cellobiose concentrations at 10 g/L intervals between 10 and 100 g/L were assessed to determine the optimal concentration for lipid and GLA production. After inoculation, the coculturing medium was incubated for 96 h at 28 °C with agitation at 150 rpm before determining fungal biomass, lipid, and GLA yields.

#### Effect of different nitrogen sources on CDW, lipid, and GLA production

Six organic nitrogen sources (ammonium tartrate, yeast extract, peptone, malt extract, urea, and casein) and three inorganic nitrogen sources (sodium nitrate, ammonium chloride, and ammonium sulfate) were assessed separately at concentrations of 1.5 and 3.3 g/L, respectively. The coculturing fermentation medium was inoculated with both organisms and incubated for 96 h at 28 °C with an agitation speed of 150 rpm before determining CDW, lipid, and GLA yields.

#### Effect of nitrogen concentration on CDW, lipid, and GLA production

Different ammonium sulfate (inorganic nitrogen) concentrations at 2–3 g/L intervals between 2.1 g/L and 2.8 g/L were assessed. After its addition, the coculturing medium was incubated under the same conditions stated above before determining the CDW, lipid, and GLA yields.

### Effect of environmental conditions

Based on the observation that biomass, lipid, and GLA yields were highest with cellobiose and ammonium sulfate, we selected these as our carbon and nitrogen sources, respectively, to determine the maximum biomass, lipid, FAs, and GLA yields of *M. plumbeus* and *B. subtilis* cocultures under different abiotic factors. 150 mL of K&R fermentation medium in 500 mL flasks was used for these experiments. Four different abiotic factors were used to assess the CDW, lipids, and GLA yields of coculture fermentation. The temperature was varied between 15 ± 2 and 40 ± 2 °C, the pH was varied between 4 and 8, the incubation time was varied between 1 and 6 days, and the agitation speed was varied between 100 and 250 rpm to determine their effects.

### Lipid extraction and fatty acid methyl ester (FAME) analysis

The extraction of lipids from the obtained dried biomass was carried out according to the Folch method with minor modifications^1^. In brief, 2 mL of 4 M hydrochloric acid was added to freeze-dried biomass (10–20 mg) and homogenised. The tubes containing acidic suspensions were heated in a water bath for 5 h at 80 °C and then vortexed 4–5 times. The mixture was cooled for 15–20 min at room temperature before the addition of 2 mL chloroform, 1 mL methanol, and 100 μL pentadecanoic acid (C15:0, internal standard) and vortexing for 30–60 s, followed by proper mixing in a vertical 360 tube rotator for 60 min. Finally, all tubes were centrifuged for 5 min at 3000 rpm to separate the two layer phases. The bottom layer phase containing the extracted lipids in 2 mL chloroform was extracted and transferred to new weighted tubes and completely evaporated under a stream of nitrogen gas before being weighted to determine total lipids mass.

### Gas chromatography (GC) analysis of FAMEs

The FA composition of the collected samples was determined via FAMEs with GC (HP 5890; Hewlett Packard, Palo Alto, CA, USA) equipped with a 30 cm 0.32 µm capillary column (BPX 70; Fisher Scientific, Waltham, MA, USA). 1 mL of anhydrous methanol (10%, in hydrochloric acid) was mixed with the extracted lipids in a water bath for 3 h at 60 °C before vortexing 2–3 times. To extract the methyl esters, 2 mL hexane and 1 mL saturated sodium chloride was added before vortexing for 30 s, and the tubes were agitated for 60 min on the vertical 360 tube rotator before being centrifuged for 5 min at 3000 rpm. The upper hexane layer was analysed by GC for FAs analysis. The GC working conditions were as follows: The injector was maintained for 3 min at 120 °C, then increased to 200 °C at 5 °C/min. Helium gas was used as a carrier when 1 µL of individual samples were injected, with a gas flow rate of 40 cm^3^ min^-1^. Continuous increasing of temperature to 210 °C at 4 °C/min with a 5 min hold, then to 220 °C with a 2 min hold. The FA chromatographic peaks were detected and identified using the Chrome Leon chromatography software (Dionex, Sunnyvale, CA, USA). Identification and quantification of individual chromatographic peaks was carried out by comparing their peak areas and retention time to the Supelco 37 external FAMEs standard mixture (Component FAME Mix; Sigma-Aldrich, St. Louis, MO, USA).

### Cultivation in 5 L fermenter

Cocultures of *M. plumbeus* and *B. subtilis* were assessed in a 5 L bench-top fermentor (New Brunswick; Eppendorf, Hamburg, Germany) with a 3 L working volume to determine the reproducibility of shaker flask fermentation data under optimal nutritional and environmental parameters. The optimal parameters identified for carbon and nitrogen sources, pH, temperature, agitation speed, and incubation periods were used with the fermentor. The fermentation media was inoculated with 10% (v/v) of seed culture of both strains, with the bacteria added 48 h after the fungus. We used aeration at 1 vvm min^−1^, and sterile polypropylene glycol as an anti-foaming agent added manually when necessary. Samples were collected every 24 h over a 144 h period to determine CDW, lipid, GLA, and FA yields.

### Statistical analysis

All the experiments were conducted in triplicate, and data are reported as the mean ± standard deviation (SD) for each group (*n* = 3). The Student’s t-test and analysis of variance (ANOVA) were performed using the OriginPro program v.95E (Origin Lab Corporation, Northampton, MA, USA). *P* < 0.05 was considered significantly significant.

### Ethics approval

This article does not contain any studies conducted by any of the authors with human or animal participants.

## Supplementary Information

Supplementary Information.

## Data Availability

The datasets generated and/or analyzed during the current study are available in the NCBI repository, https://blast.ncbi.nlm.nih.gov/Blast.cgi#alnHdr_1846564652, as (*M. plumbeus* strain AUMC 6697.A) under GenBank accession number: MT539120, and *B. subtilis* bacteria at https://www.atcc.org/ with ATCC ID: ATCC 6633.

## References

[1] Shah AM (2021). Isolation, characterization and fatty acid analysis of *Gilbertella persicaria* DSR1: A potential new source of high value single-cell oil. Biomass Bioenergy.

[2] Mohamed H (2020). Comparative analysis of different isolated Oleaginous Mucoromycota fungi for their γ-linolenic acid and carotenoid production. BioMed Res. Int..

[3] Ghazani SM, Marangoni AG (2022). Microbial lipids for foods. Trends Food Sci. Technol..

[4] Linder T (2019). Making the case for edible microorganisms as an integral part of a more sustainable and resilient food production system. Food Secur..

[5] Kirrolia A, Bishnoi NR, Singh R (2013). Microalgae as a boon for sustainable energy production and its future research & development aspects. Renew. Sustain. Energy Rev..

[6] Matsungo TM, Siziba LP, Galanakis CM (2020). Chapter 1—Lipids and nutrition security. Lipids and Edible Oils.

[7] Marmann A (2014). Co-cultivation—A powerful emerging tool for enhancing the chemical diversity of microorganisms. Mar. Drugs.

[8] Pfannenstiel BT, Keller NP (2019). On top of biosynthetic gene clusters: How epigenetic machinery influences secondary metabolism in fungi. Biotechnol. Adv..

[9] Yen HW, Chen PW, Chen LJ (2015). The synergistic effects for the co-cultivation of oleaginous yeast—*Rhodotorula glutinis* and microalgae—*Scenedesmus obliquus* on the biomass and total lipids accumulation. Biores. Technol..

[10] Feng Y (2021). Enhancement of lipid productivity and self-flocculation by cocultivating *Monoraphidium* sp. FXY-10 and *Heveochlorella* sp. Yu under mixotrophic mode. Appl. Biochem. Biotechnol..

[11] Brakhage AA, Schroeckh V (2011). Fungal secondary metabolites—Strategies to activate silent gene clusters. Funct. Gene Biol..

[12] Abdel-Wahab NM (2019). Induction of secondary metabolites from the marine-derived fungus *Aspergillus versicolor* through co-cultivation with *Bacillus subtilis*. Planta. Med..

[13] Jiang Y (2019). Recent advances of biofuels and biochemicals production from sustainable resources using co-cultivation systems. Biotechnol. Biofuels.

[14] Liu S (2016). Cytotoxic 14-membered macrolides from a mangrove-derived endophytic fungus, *Pestalotiopsis microspora*. J. Nat. Prod..

[15] Ebrahim W (2016). Metabolites from the fungal endophyte *Aspergillus austroafricanus* in axenic culture and in fungal–bacterial mixed cultures. J. Nat. Prod..

[16] Cheirsilp B, Suwannarat W, Niyomdecha R (2011). Mixed culture of oleaginous yeast *Rhodotorula glutinis* and microalga *Chlorella vulgaris* for lipid production from industrial wastes and its use as biodiesel feedstock. Nat. Biotechnol..

[17] Magdouli S, Brar SK, Blais JF (2016). Co-culture for lipid production: Advances and challenges. Biomater. Bioenergy.

[18] Akone SH (2016). Inducing secondary metabolite production by the endophytic fungus *Chaetomium* sp. through fungal–bacterial co-culture and epigenetic modification. Tetrahedron.

[19] Toyama T (2019). Enhanced production of biomass and lipids by *Euglena gracilis* via co-culturing with a microalga growth-promoting bacterium, *Emticicia* sp. EG3. Biotechnol. Biofuels.

[20] Patel A (2020). An overview of potential Oleaginous microorganisms and their role in biodiesel and omega-3 fatty acid-based industries. Microorganisms..

[21] Patra JK (2016). Kimchi and other widely consumed traditional fermented foods of Korea: A review. Front. Microbiol..

[22] Khalil ZG (2019). Inter-Kingdom beach warfare: Microbial chemical communication activates natural chemical defences. ISME J..

[23] Baral B, Akhgari A, Metsä-Ketelä M (2018). Activation of microbial secondary metabolic pathways: Avenues and challenges. Synth. Syst. Biotechnol..

[24] Zhao H (2021). High-yield oleaginous fungi and high-value microbial lipid resources from Mucoromycota. Bioenergy Res..

[25] Shah AM (2022). Microbes: A hidden treasure of polyunsaturated fatty acids. Front. Nutr..

[26] Peng XY (2021). Co-culture: stimulate the metabolic potential and explore the molecular diversity of natural products from microorganisms. Mar. Life Sci. Technol..

[27] Wakefifield J (2017). Dual induction of new microbial secondary metabolites by fungal bacterial co-cultivation. Front. Microbiol..

[28] Yu G (2021). Coculture, an efficient biotechnology for mining the biosynthesis potential of macrofungi via interspecies interactions. Front. Microbiol..

[29] Anittaya K, Prapassorn DE, Ekachai C (2017). Fatty acid profiles of fermented soybean prepared by *Bacillus subtilis* and *Rhizopus oligosporus*. Environ. Exp. Biol..

[30] Nugroho WA (2015). Efect of growth promoting bacteria on the growth rate and lipid content of microalgae Chorella sp. in sludge liquor of anaerobic digester of dairy manure. Int. J. Adv. Sci. Eng. Inf. Technol..

[31] Mohamed H (2017). Production of phytotoxic polyketide spiciferone a by *Phoma fungicola*. Mikol. Fitopatol..

[32] Gupta S, Bhatt P, Chaturvedi P (2018). Determination and quantification of asiaticoside in endophytic fungus from *Centella asiatica* (L.) Urban. World J. Microbiol. Biotechnol..

[33] Kitcha S, Cheirsilp B (2014). Enhanced lipid production by co-cultivation and co-encapsulation of oleaginous yeast *Trichosporonoides spathulata* with microalgae in alginate gel beads. Appl. Biochem. Biotechnol..

[34] Liu G (2022). *Bacillus subtilis* BS-15 effectively improves Plantaricin production and the regulatory biosynthesis in *Lactiplantibacillus plantarum* RX-8. Front. Microbiol..

[35] Wenjun B (2021). Approaches to improve the lipid synthesis of oleaginous yeast *Yarrowia lipolytica*: A review. Renew. Sustain. Energy Rev..

[36] Gao B (2021). Biomass, lipid accumulation kinetics, and the transcriptome of heterotrophic oleaginous microalga *Tetradesmus bernardii* under different carbon and nitrogen sources. Biotechnol. Biofuels..

[37] Yao Q (2019). An efficient strategy for screening polyunsaturated fatty acid-producing oleaginous filamentous fungi from soil. J. Microbiol. Methods.

[38] Benjamas C, Suleeporn K, Salwa T (2012). Co-culture of an oleaginous yeast *Rhodotorula glutinis* and a microalga *Chlorella vulgaris* for biomass and lipid production using pure and crude glycerol as a sole carbon source. Ann. Microbiol..

[39] Lopes HJS (2020). C/N ratio and carbon source-dependent lipid production profiling in *Rhodotorula toruloides*. Appl. Microbiol. Biotechnol..

[40] Rasmey AM, Tawfik MA, Abdel-Kareem MM (2020). Direct transesterification of fatty acids produced by *Fusarium solani* for biodiesel production: Effect of carbon and nitrogen on lipid accumulation in the fungal biomass. J. Appl. Microbiol..

[41] Karim A (2021). Yeast and bacteria co-culture-based lipid production through bioremediation of palm oil mill effluent: A statistical optimization. Biomass Conv. Bioref..

[42] Cai S, Hu C, Du S (2007). Comparisons of growth and biochemical composition between mixed culture of alga and yeast and monocultures. J. Biosci. Bioeng..

[43] Xue F (2010). A new strategy for lipid production by mix cultivation of *Spirulina platensis* and *Rhodotorula glutinis*. Appl. Biochem. Biotechnol..

[44] Cheirsilp B, Kitcha S, Torpee SJ (2012). Co-culture of an oleaginous yeast *Rhodotorula glutinis* and a microalga *Chlorella vulgaris* for biomass and lipid production using pure and crude glycerol as a sole carbon source. Ann. Microbiol..

[45] Zhao P (2014). Enhancing lipid productivity by co-cultivation of *Chlorella* sp. U4341 and *Monoraphidium* sp. FXY-10. J. Biosci. Bioeng..

[46] Cheirsilp B, Suwannarat W, Niyomdecha R (2011). Mixed culture of oleaginous yeast *Rhodotorula glutinis* and microalga *Chlorella vulgaris* for lipid production from industrial wastes and its use as biodiesel feedstock. N. Biotechnol..

[47] Shoaib A (2018). Optimization of cultural conditions for lipid accumulation by *Aspergillus wentii* Ras101 and its transesterification to biodiesel: Application of response surface methodology. 3 Biotech..

[48] Abdelhamid SA (2019). Optimization of culture conditions for biodiesel production from Egyptian isolate *Penicillium commune* NRC2016. Bull. Nat. Res. Cent..

[49] Wang Y (2015). Optimization of Chlorella vulgaris and bioflocculant producing bacteria co-culture: Enhancing microalgae harvesting and lipid content. Lett. Appl. Microbiol..

[50] Mamatha SS, Ravi R, Venkateswaran G (2008). Medium optimization of gamma linolenic acid production in *Mucor rouxii* CFR -G15 using RSM. Food Bioprocess Technol..

[51] Xian M (2002). Production of linolenic acid by *Mortierella isabellina* grown on octadecanol. Curr. Microbiol..

[52] Nosheen S (2021). Role of Snf-β in lipid accumulation in the high lipid-producing fungus *Mucor circinelloides* WJ11. Microb. Cell Fact..

[53] Naz T (2020). Redirecting metabolic flux towards the mevalonate pathway for enhanced β-carotene production in *M. circinelloides* CBS 277.49. BioMed Res. Int..

