# Supplementary material for: Coculturing of Mucor plumbeus and Bacillus subtilis bacterium as an efficient fermentation strategy to enhance fungal lipid and gamma-linolenic acid (GLA) production
Source: Sci Rep. 2022 Jul 30;12:13111. doi: 10.1038/s41598-022-17442-2 (PMC9338991; doi:10.1038/s41598-022-17442-2)
Supplement: Supplementary file 1 — Supplementary Information. [file 41598_2022_17442_MOESM1_ESM.docx]

**Supplementary material**

**Table S1.** List of Mucoromycota fungal strains used in this study

| **No.** | **Name** | **Strain code** | **Accession number** | **Isolation source** |
| --- | --- | --- | --- | --- |
| 1 | *Rhizomucor pusillus* | AUMC 11616.A | MT509982 | Cattle manure |
| 2 | *Mucor circinelloides* | AUMC 698 | MT365786 | Onion, Cairo |
| 3 | *M. circinelloides* | AUMC 697 | MT365787 | Soil, Alexandria |
| 4 | *M. circinelloides* | AUMC 11641 | MT365790 | Soil, Assiut |
| 5 | *M. circinelloides* | AUMC 6027 | MT365788 | Cow dung |
| 6 | *M. hiemalis* | AUMC 6695 | MT365793 | Textile 100% polyester |
| 7 | *M. circinelloides* | AUMC 6696.A | MT509983 |  |
| 8 | *plumbeus* | AUMC 6697.A | MT539120 | Textile polyester/cotton (1:1) |
| 9 | *M. hiemalis* | AUMC 6031 | MT365791 | Horse dung |
| 10 | *M. hiemalis* | AUMC 6036 | MT365792 | Clove plants |
| 11 | *M. hiemalis* | AUMC 9172 | MT365796 | larynx specimen, Assiut |
